# Hydroxychloroquine-induced cytotoxicity in ARPE-19 cells involves PI3K/AKT-associated signaling: network toxicology and functional validation

**DOI:** 10.3389/fphar.2026.1917634

**Published:** 2026-10-26

**Authors:** Lan Lin, Yunhao Zhou, Guanghui Liu, Songjie Lu, Ting Huang, Zhaoyang Xu, Sheng Chen

**Affiliations:** 1 Department of Ophthalmology, The Second Affiliated Hospital of Fujian University of Traditional Chinese Medicine, Fuzhou, China; 2 Department of Bioengineering, College of Biological Science and Biotechnology, Fuzhou University, Fuzhou, China; 3 Department of Ophthalmology, Affiliated People’s Hospital (Fujian Provincial People’s Hospital), Fujian University of Traditional Chinese Medicine, Fuzhou, China; 4 Department of Ophthalmology, Longhua Hospital Affiliated to Shanghai University of Traditional Chinese Medicine, Shanghai, China

**Keywords:** hydroxychloroquine, network toxicology, PI3K/AKT signaling pathway, retinal toxicity, SC79

## Abstract

**Background:**

Hydroxychloroquine (HCQ) is widely used for the long-term treatment of autoimmune and rheumatologic disorders, but retinal toxicity remains an important adverse effect. Although lysosomal and autophagic dysfunction have been implicated in HCQ-associated retinal injury, the signaling networks involved in the cellular response to HCQ remain incompletely understood. This study investigated the potential involvement of PI3K/AKT-associated signaling in HCQ-induced cytotoxicity.

**Methods:**

Network toxicology and molecular docking were integrated to identify candidate HCQ-associated targets, followed by experimental validation in human retinal pigment epithelial ARPE-19 cells. Cell viability was evaluated by CCK-8 assay, and apoptosis was assessed by Annexin V/PI flow cytometry. The expression of predicted hub genes and proteins was examined by qRT-PCR and Western blotting. AKT phosphorylation was evaluated across HCQ concentration and time gradients. The AKT activator SC79 was further used in pharmacological rescue experiments to assess the functional involvement of AKT signaling in HCQ-induced apoptosis.

**Results:**

Network analysis identified PIK3CA, PIK3R1, SRC, JAK2, and PTPN11 as candidate hub proteins associated with PI3K/AKT-related signaling. Molecular docking suggested potential interactions between HCQ and these proteins, with variable docking energies among targets. HCQ reduced ARPE-19 cell viability in a concentration-dependent manner, with a 24-h IC_50_ of 59.51 μM, and increased apoptosis with increasing concentrations. qRT-PCR revealed concentration-dependent and gene-specific transcriptional responses rather than uniform suppression of all five hub genes, whereas Western blotting showed an overall reduction in the abundance of the candidate hub proteins after 24 h HCQ exposure. AKT phosphorylation exhibited dynamic regulation: short-term HCQ exposure increased p-AKT/AKT at higher concentrations, whereas an independent 30 μM time-course experiment showed an overall decline in AKT phosphorylation during prolonged exposure. Importantly, SC79 pretreatment enhanced p-AKT/AKT under HCQ exposure and partially attenuated HCQ-induced apoptosis.

**Conclusion:**

These findings support the involvement of PI3K/AKT-associated signaling in HCQ-induced cytotoxicity in ARPE-19 cells. HCQ induces concentration- and time-dependent alterations in this signaling network, while pharmacological enhancement of AKT signaling partially reduces the apoptotic response. The results provide functional evidence that AKT signaling contributes to the cellular response to HCQ and support further investigation of PI3K/AKT-associated signaling in HCQ-related RPE injury.

## 1 Introduction

Hydroxychloroquine (HCQ), a 4-aminoquinoline derivative of chloroquine, is widely used as an antimalarial and immunomodulatory agent for systemic autoimmune diseases, including rheumatoid arthritis and systemic lupus erythematosus (Al-Hamadani et al., 2025). HCQ has a prolonged elimination half-life and extensive tissue distribution, including accumulation in melanin-containing tissues such as the retina (Rao et al., 2023). Among its long-term adverse effects, HCQ retinopathy is of particular clinical concern because established retinal damage can lead to irreversible visual impairment (Eljaaly et al., 2020; Choudhuri et al., 2021; Dey et al., 2023).

HCQ retinopathy represents an important long-term safety concern in patients receiving chronic therapy (Melles et al., 2023). Clinical studies indicate that early retinal alterations predominantly involve the outer retina and photoreceptor layers and may precede overt structural changes in the retinal pigment epithelium (RPE) (de Sisternes et al., 2015). Current screening strategies rely on multimodal structural and functional assessments, including optical coherence tomography (OCT), fundus autofluorescence (FAF), and visual-field testing (Rosenbaum et al., 2021).

Although advances in ophthalmic imaging have improved the detection of HCQ-associated retinal abnormalities, these approaches primarily characterize structural and functional manifestations of toxicity and do not directly define the molecular signaling events associated with HCQ-induced cellular injury. The molecular networks linking HCQ exposure to RPE cellular dysfunction and apoptosis remain incompletely understood. Further clarification of these molecular responses may therefore provide additional insight into the cellular mechanisms underlying HCQ-associated RPE injury.

To address this question, the present study integrated network toxicology and molecular docking to identify candidate molecular targets and signaling pathways associated with HCQ-related retinal toxicity, followed by experimental validation in human ARPE-19 cells. We evaluated HCQ-induced changes in cell viability and apoptosis, examined the transcriptional and protein-level responses of predicted hub targets, and characterized concentration- and time-dependent alterations in AKT phosphorylation. In addition, pharmacological rescue experiments using the AKT activator SC79 were performed to assess the functional involvement of AKT signaling in the cellular response to HCQ. Through this integrated computational and experimental approach, we sought to characterize the potential involvement of PI3K/AKT-associated signaling in HCQ-induced cytotoxicity in ARPE-19 cells.

## 2 Methods

### 2.1 Identification of HCQ-related targets

The chemical structure and canonical SMILES format of HCQ were acquired from the PubChem database (Kim et al., 2025). These identifiers were utilized to predict potential pharmacological targets using the SwissTargetPrediction (Daina et al., 2019), PharmMapper (Wang et al., 2017), and TargetNet databases (Yao et al., 2016). Target gene names were subsequently standardized via the UniProt Knowledgebase (UniProtKB) (The UniProt Consortium, 2025). After merging the datasets and eliminating duplicates, a comprehensive HCQ target library was constructed.

### 2.2 Retrieval of retinal toxicity-associated targets

Disease-associated targets were mined from the DisGeNET (Piñero et al., 2017), OMIM(Hamosh, 2004), and GeneCards databases (Safran et al., 2010) using the keyword “retinal toxicity”. The nomenclature of the retrieved genes and proteins was normalized using the UniProtKB database (The UniProt Consortium et al., 2025) to ensure consistency. Following the removal of duplicate entries, a unified dataset of retinal toxicity-associated targets was generated.

### 2.3 Identification of intersecting targets

To pinpoint the potential toxicological targets of HCQ in the retina, the HCQ-related targets and the retinal toxicity-associated targets were intersected. An intersection Venn diagram was generated using the “VennDiagram” package in R software (v4.5.3) (Chen and Boutros, 2011; R Core Team, 2023).

### 2.4 Construction of the protein-protein interaction (PPI) network

The intersecting genes shared by HCQ and retinal toxicity were mapped into the STRING database (Szklarczyk et al., 2023) to evaluate protein-protein interactions (PPIs). The organism was restricted to “*Homo sapiens*”, and the minimum required interaction score was set to >0.7 (high confidence). The resultant TSV files were exported and visualized using Cytoscape software. To identify the core hub targets within the biological network, topological analysis was performed using the CytoHubba plugin (Chin et al., 2014) to screen for key nodes.

### 2.5 GO and KEGG enrichment analyses

To elucidate the molecular mechanisms underlying HCQ-induced retinal toxicity, the intersecting target genes were first converted into Entrez IDs using the “org.Hs.eg.db” package in R software (v4.5.3) (R Core Team, 2023; Carlson, 2024). Subsequently, Gene Ontology (GO) functional annotation (The Gene Ontology Consortium, 2026) and Kyoto Encyclopedia of Genes and Genomes (KEGG) pathway enrichment analyses (Kanehisa and Sato, 2026) were conducted utilizing the “clusterProfiler” package (Yu et al., 2012). Results were considered statistically significant if both the *P*-value and the adjusted *P*-value (*q*-value) were <0.05. The data were visualized using the “enrichplot” (Yu, 2026)and “ggplot2″ packages (Hadley, 2016), displaying the top six significantly enriched GO terms and the top 20 KEGG pathways ranked by ascending *q*-values.

### 2.6 Molecular docking simulation

Molecular docking was performed to investigate the potential interactions between hydroxychloroquine (HCQ) and the identified core hub proteins. The three-dimensional structure of HCQ was obtained from the PubChem database (Kim et al., 2025), whereas the crystal structures of the target proteins were retrieved from the RCSB Protein Data Bank according to their corresponding UniProt entries. The receptor structures used for docking were PIK3CA (PDB ID: 8EXL), PIK3R1 (PDB ID: 7RNS), SRC (PDB ID: 2BDJ), JAK2 (PDB ID: 7RN6), and PTPN11 (PDB ID: 3B7O). Protein structures were selected based on species, structural quality, absence of relevant mutations, and the availability of experimentally characterized functional or ligand-binding regions.

Prior to docking, receptor and ligand structures were prepared using AutoDockTools 1.5.7. Crystallographic water molecules, non-relevant heteroatoms, and co-crystallized ligands were removed from the receptor structures, followed by the addition of polar hydrogen atoms and assignment of Gasteiger charges. HCQ was prepared as a flexible ligand by assigning Gasteiger charges and defining rotatable bonds. Molecular docking was subsequently performed using AutoDock 4.2.6 with the Lamarckian genetic algorithm. For each receptor, the grid box was positioned to encompass the known functional or ligand-binding pocket, with the binding site of the co-crystallized ligand used as a structural reference when available. The genetic algorithm was performed with a population size of 150, a maximum of 2.5 × 10^6 energy evaluations, 27,000 generations, and 10 independent docking runs. The resulting docking conformations were clustered using a root-mean-square deviation (RMSD) tolerance of 2.0 Å.

For each target protein, the pose with the lowest AutoDock-estimated docking energy within the functionally relevant binding region was selected for subsequent analysis. The AutoDock-derived energies were interpreted as computational docking scores rather than experimentally determined binding affinities. Representative protein–HCQ complexes were visualized in three dimensions using PyMOL 3.0.3. Protein–ligand interactions, including hydrogen bonds and surrounding interacting residues, were further examined and illustrated using Maestro (Schrödinger, 2021).

### 2.7 Cell culture

ARPE-19 cells, obtained from Shanghai Fuyu Biotechnology Co., Ltd. (Shanghai, China), were cultured in Dulbecco’s Modified Eagle Medium/Nutrient Mixture F-12 (DMEM/F-12; Biosharp, China) supplemented with 10% fetal bovine serum (FBS; Gibco) and 1% penicillin–streptomycin (Gibco). Cells at passages below 20 were used for all experiments and were confirmed to be free of *mycoplasma* contamination. Cells were maintained at 37 °C in a humidified incubator containing 5% CO_2_.

### 2.8 HCQ solution preparation

Hydroxychloroquine free base (HCQ; CAS No. 118–42-3; purity ≥98%) was obtained from Aladdin (Shanghai, China). HCQ was dissolved in dimethyl sulfoxide (DMSO; Sigma-Aldrich) to prepare a stock solution and subsequently diluted with culture medium to the required experimental concentrations. An equivalent volume of DMSO was added to the vehicle control group, and the final DMSO concentration was maintained below 0.1% (v/v) in all groups.

### 2.9 CCK-8 assay

ARPE-19 cells were seeded into 96-well plates at a density of 5 × 10^3^ cells/well and exposed to HCQ at concentrations of 0, 15, 30, 45, 60, 75, 90, 120, and 150 μM for 24 h. Each concentration was tested in five technical replicate wells, and the experiment was independently repeated three times. The five technical replicate wells within each independent experiment were averaged to generate a single biological replicate value for each concentration. Following HCQ exposure, 10 μL of CCK-8 reagent (Adamas Life, 04483230, China) was added to each well, followed by incubation for 2 h at 37 °C. Absorbance was measured at 450 nm using a Synergy H1 multimode microplate reader (BioTek, USA). Cell viability was normalized to the vehicle control. The 24-h IC_50_ was estimated by nonlinear regression using a log (inhibitor) versus normalized response model with a variable slope.

Based on the 24-h concentration–response curve and the estimated IC50, HCQ concentrations of 15, 30, and 60 μM were selected for subsequent mechanistic experiments to represent low, intermediate, and high exposure concentrations, respectively. The highest concentration was selected to approximate the estimated IC50, whereas the two lower concentrations represented progressively lower sub-IC50 exposure levels.

### 2.10 Quantitative real-time polymerase chain reaction (qRT-PCR)

ARPE-19 cells were treated with 0, 15, 30, or 60 μM HCQ for 24 h. A total of 1 μg of RNA was used for each reverse-transcription reaction. First-strand cDNA was synthesized using the SPARKscript II All-in-one RT SuperMix for qPCR with gDNA Eraser (SparkJade, AG0305-B, China). Each 20-μL reverse-transcription reaction contained total RNA, 1 μL gDNA Eraser, 4 μL 5× SPARKscript II All-in-one qRT SuperMix, and RNase-free water. Reverse transcription was performed at 50 °C for 15 min, followed by 85 °C for 5 s.

Quantitative real-time PCR was performed using the 2× Universal SYBR Green qPCR Mix (SparkJade, AH0105-B, China) on a SLAN-96S Real-Time PCR System (Hongshi, China). Each 20-μL reaction contained 10 μL 2× Universal SYBR Green qPCR Mix, 0.4 μL each of forward and reverse primers (10 μM), cDNA template, and RNase-free water. The amplification program consisted of initial denaturation at 95 °C for 30 s, followed by 40 cycles of denaturation at 95 °C and annealing/extension at 60 °C, followed by melt-curve analysis to verify amplification specificity. The relative mRNA expression levels of *PIK3CA*, *PIK3R1*, *SRC*, *JAK2*, and *PTPN11* were determined.

*GAPDH* and *RPLP0* were jointly used as reference genes. For each biological sample, the arithmetic mean of the Ct values of GAPDH and RPLP0 was used as the composite reference Ct, and relative gene expression was calculated using the 2^−ΔΔCt method with the vehicle control group (0 μM HCQ) as the calibrator. Three independent biological experiments were performed. The primer sequences and corresponding amplification characteristics are provided in Table 1.

**TABLE 1:** Primer information used for qRT-PCR.

Primer name	RefSeq accession	Primer sequence (5′–3′)	Exon information	Amplification efficiency (%)	Amplicon size (bp)
PIK3CA-F	NM_006218.4	TGT​TAC​TCA​AGA​AGC​AGA​AAG​GGA​AG	Exon 2	93.2	109
PIK3CA-R		ACG​GTT​GCC​TAC​TGG​TTC​AAT​TAC	Exon 2		
PIK3R1-F	NM_181523.3	GAA​GCA​GCA​ACC​GAA​ACA​AAG​C	Exon 15	98.4	109
PIK3R1-R		ACC​ACT​ACA​GAG​CAG​GCA​TAG​C	Exon 15–16 junction		
SRC-F	NM_005417.5	TCC​AAG​CCG​CAG​ACT​CAG​G	Exon 7	110.6	108
SRC-R		CAT​CCA​CAC​CTC​GCC​AAA​GC	Exon 7		
JAK2-F	NM_004972.4	ACT​GAA​GAG​CAC​CTA​AGA​GAC​TTT​G	Exon 20	108.2	108
JAK2-R		ACG​CCG​ACC​AGC​ACT​GTA​G	Exon 20–21 junction		
PTPN11-F	NM_002834.5	GCG​GTC​CAG​CAT​TAT​ATT​GAA​ACA​C	Exon 13	111.3	127
PTPN11-R		GGC​TCT​GAT​CTC​CAC​TCG​TCT​G	Exon 14		
GAPDH-F	NM_002046.7	GGT​GTG​AAC​CAT​GAG​AAG​TAT​GA	Exon 6	102.4	123
GAPDH-R		GAG​TCC​TTC​CAC​GAT​ACC​AAA​G	Exon 7		
RPLP0-F	NM_001002.4	GCA​GCA​TCT​ACA​ACC​CTG​AAG	Exon 6	102.6	87
RPLP0-R		CAC​TGG​CAA​CAT​TGC​GGA​C	Exon 7		

### 2.11 Western blotting

ARPE-19 cells were exposed to HCQ at concentrations of 0, 15, 30, or 60 μM for 24 h to assess the protein abundance of the predicted hub targets PIK3CA, PIK3R1, SRC, JAK2, and PTPN11. To evaluate HCQ-associated changes in AKT phosphorylation, cells were treated with 0, 15, 30, or 60 μM HCQ for either 1 or 24 h. In addition, a time-course experiment was performed by treating ARPE-19 cells with 30 μM HCQ for 0, 1, 3, 6, 12, or 24 h.

Following treatment, cells were washed with ice-cold PBS and lysed in RIPA buffer supplemented with 1% protease and phosphatase inhibitor cocktail for 30 min on ice. Protein concentrations were determined using a BCA protein assay kit (A55864; Thermo Fisher Scientific, USA). Equal amounts of total protein (15 μg per lane) were mixed with 5× SDS-PAGE loading buffer and heated at 100 °C for 10 min. Protein samples were separated using 12.5% SDS-PAGE gels and subsequently transferred onto 0.45-μm PVDF membranes (Merck, Germany) at a constant current of 200 mA for 65 min.

Following transfer, the membranes were washed with 1× TBST and blocked for 1 h at room temperature with 5% non-fat milk or 5% bovine serum albumin (BSA), as appropriate. For phospho-AKT detection, membranes were blocked with 5% BSA, whereas membranes used for detection of total proteins were blocked with 5% non-fat milk. The membranes were then incubated overnight at 4 °C with the following primary antibodies: anti-PIK3CA (1:2,000, Cat#67071-1-Ig), anti-PIK3R1 (1:10,000, Cat#60225-1-Ig), anti-SRC (1:1,000, Cat#25978-1-AP), and anti-PTPN11 (1:10,000, Cat#82503-1-RR) from Proteintech (China); and anti-JAK2 (1:1,000, Cat#3230), anti-AKT (1:1,000, Cat#9272), anti-phospho-AKT (Ser473) (1:1,000, Cat#4060), and anti-β-actin (1:1,000, Cat#4970) from Cell Signaling Technology (USA).

After three washes with 1× TBST, the membranes were incubated for 1 h at room temperature with HRP-conjugated goat anti-rabbit IgG (1:3,000, Cat#7074P2) or goat anti-mouse IgG (1:3,000, Cat#91196) secondary antibodies (Cell Signaling Technology, USA), as appropriate. Following additional washes with TBST, immunoreactive bands were visualized using an enhanced chemiluminescence (ECL) detection reagent and captured using a chemiluminescence imaging system (JS-M6; Peiqing Science & Technology, China). Exposure conditions were adjusted to avoid signal saturation.

Band intensities were quantified using ImageJ software (NIH, Bethesda, MD, USA). The protein abundance of PIK3CA, PIK3R1, SRC, JAK2, and PTPN11 was normalized to β-actin. AKT phosphorylation was evaluated using the phospho-AKT/total AKT (p-AKT/AKT) ratio. Three independent biological experiments were performed for each experimental condition.

### 2.12 Annexin V–FITC/PI apoptosis assay

ARPE-19 cells were treated with HCQ at 0, 15, 30, or 60 μM for 24 h. Following treatment, both floating and adherent cells were collected, washed twice with ice-cold PBS, and resuspended in 500 μL of 1× Annexin V Binding Buffer. Cells were stained with 2 μL Annexin V-FITC and 2.5 μL propidium iodide (PI) using an Annexin V-FITC/PI apoptosis detection kit (Abbkine, KTA0006, China), followed by incubation on ice in the dark for 15 min. Samples were analyzed within 30 min using a BriCyte E6 flow cytometer (Mindray, China), with 10,000 events acquired per sample.

Unstained, Annexin V-FITC single-stained, and PI single-stained controls were used to establish fluorescence compensation and quadrant boundaries. Flow cytometry data were analyzed using FlowJo software (version 10.8.1). Viable cells were defined as Annexin V^−^/PI^−^, early apoptotic cells as Annexin V^+^/PI^−^, and late apoptotic cells as Annexin V^+^/PI^+^. Total apoptosis was calculated as the sum of the early and late apoptotic populations. Three independent biological experiments were performed.

### 2.13 SC79 treatment and AKT rescue experiments

To evaluate the functional involvement of AKT signaling, the AKT activator SC79 (purity, 98%; Cat. No. S80614; Yuanye Biotechnology, Shanghai, China) was used in pharmacological rescue experiments. SC79 was dissolved in DMSO, and the final concentration of DMSO in the culture medium was maintained below 0.1% (v/v). ARPE-19 cells were initially treated with SC79 at 0, 2.5, 5, 7.5, or 10 μg/mL to evaluate its effects on cell viability using the CCK-8 assay and on AKT phosphorylation by Western blotting. Based on these screening experiments, 5 μg/mL SC79 was selected for subsequent rescue experiments.

For the rescue experiments, ARPE-19 cells were assigned to four groups: Control, SC79, HCQ, and HCQ + SC79. In the HCQ + SC79 group, cells were pretreated with SC79 (5 μg/mL) for 1 h before exposure to HCQ (60 μM) for 24 h. The SC79 group was treated with SC79 (5 μg/mL), whereas the HCQ group was treated with HCQ (60 μM) for 24 h. AKT phosphorylation was evaluated by Western blotting as described above, and apoptosis was assessed using Annexin V–FITC/PI flow cytometry. Three independent biological experiments were performed.

### 2.14 Statistical analysis

Data are presented as mean ± standard deviation (SD) from three independent biological experiments. Technical replicates within each experiment were averaged before statistical analysis. Statistical analyses were performed using GraphPad Prism version 9.5.1. Multiple-group comparisons were performed using one-way ANOVA followed by Dunnett’s or Tukey’s multiple-comparisons test, as appropriate. The 24-h IC_50_ of HCQ was estimated by nonlinear regression using a log (inhibitor) versus normalized response model with a variable slope. A *P* value <0.05 was considered statistically significant.

## 3 Results

### 3.1 Identification of intersecting targets associated with HCQ-induced retinal toxicity

To identify the potential pharmacological targets of HCQ, we aggregated 675 HCQ-related target genes from the SwissTargetPrediction, PharmMapper, and TargetNet databases (Figure 1A). Concurrently, a total of 6,155 genes associated with retinal toxicity were retrieved from the DisGeNET, OMIM, and GeneCards databases after the removal of duplicate entries (Figure 1B). By mapping the HCQ targets against the disease-associated targets, we identified an intersection of 480 shared genes (Figure 1C). These 480 overlapping genes were considered the putative core targets mediating HCQ-induced retinal toxicity and were selected for further analysis. The detailed lists of the 480 intersecting targets supporting the findings of this study are available in Mendeley Data at doi: 10.17632/4k9p437cw8.1 (Lin et al., 2026).

**FIGURE 1:**
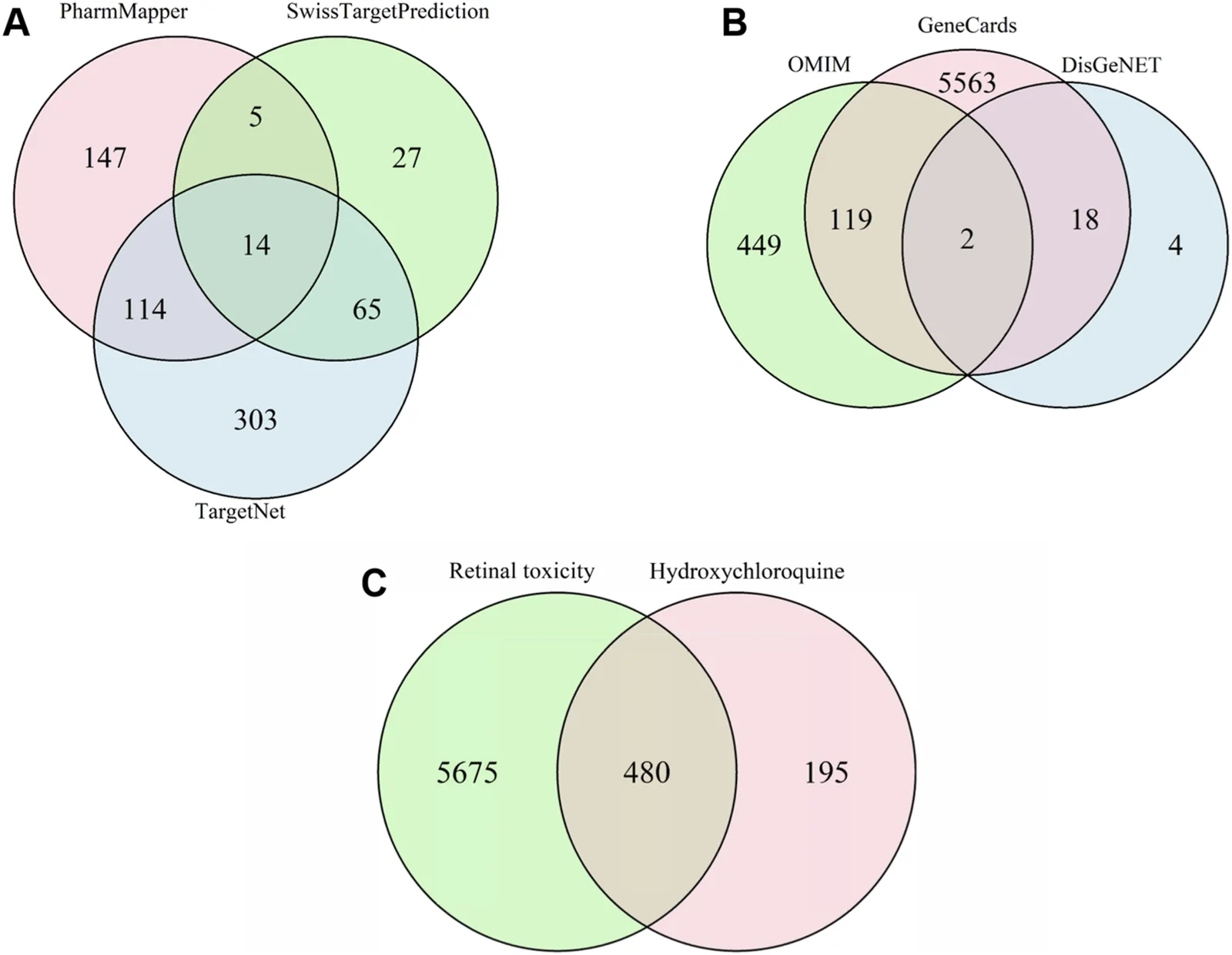
Identification of potential pharmacological targets for HCQ-induced retinal toxicity. **(A)** Venn diagram illustrating the distribution of 675 HCQ-related targets predicted by the PharmMapper, SwissTargetPrediction, and TargetNet databases. **(B)** Venn diagram showing 6,155 retinal toxicity-associated targets retrieved from the OMIM, GeneCards, and DisGeNET databases. **(C)** Intersection of HCQ-related targets and retinal toxicity-associated targets, yielding a total of 480 shared potential targets for further analysis.

### 3.2 Construction of the PPI network and identification of core targets

To elucidate the complex interactions among the 480 shared targets, a protein-protein interaction (PPI) network was constructed utilizing the STRING database. The global network comprised 480 nodes and 2,994 edges, with an average node degree of 12.5. For deeper topological analysis and visualization, the network data were imported into Cytoscape software. To pinpoint the most critical hub targets within this dense network, we utilized the CytoHubba plugin. Specifically, the Maximal Clique Centrality (MCC) algorithm—a highly robust topological scoring method for identifying essential proteins in biological networks—was applied to extract the top 20 core targets.

As depicted in the refined sub-network (Figure 2), the top 20 identified hub targets included *PIK3CA*, *PIK3R1*, *SRC*, *JAK2, PTPN11*, *EGFR*, *FYN*, *PIK3CB*, *PTK2*, *PIK3CG*, *LYN*, *GRB2*, *PIK3CD*, *AKT1*, *PDGFRB*, *ERBB2*, *HCK*, *STAT1*, *KDR*, and *PDGFRA*. Notably, *PIK3CA*, *PIK3R1*, *SRC*, *JAK2*, and *PTPN11* exhibited the highest MCC connectivity scores, visually distinguished by the dark red nodes in the diagram. These top-ranking hub targets are hypothesized to act as the primary molecular nodes mediating HCQ-induced retinal cytotoxicity, thereby establishing the foundation for our subsequent *in vitro* validation. Detailed topological parameters for the top 20 core targets are listed in Table 2.

**FIGURE 2:**
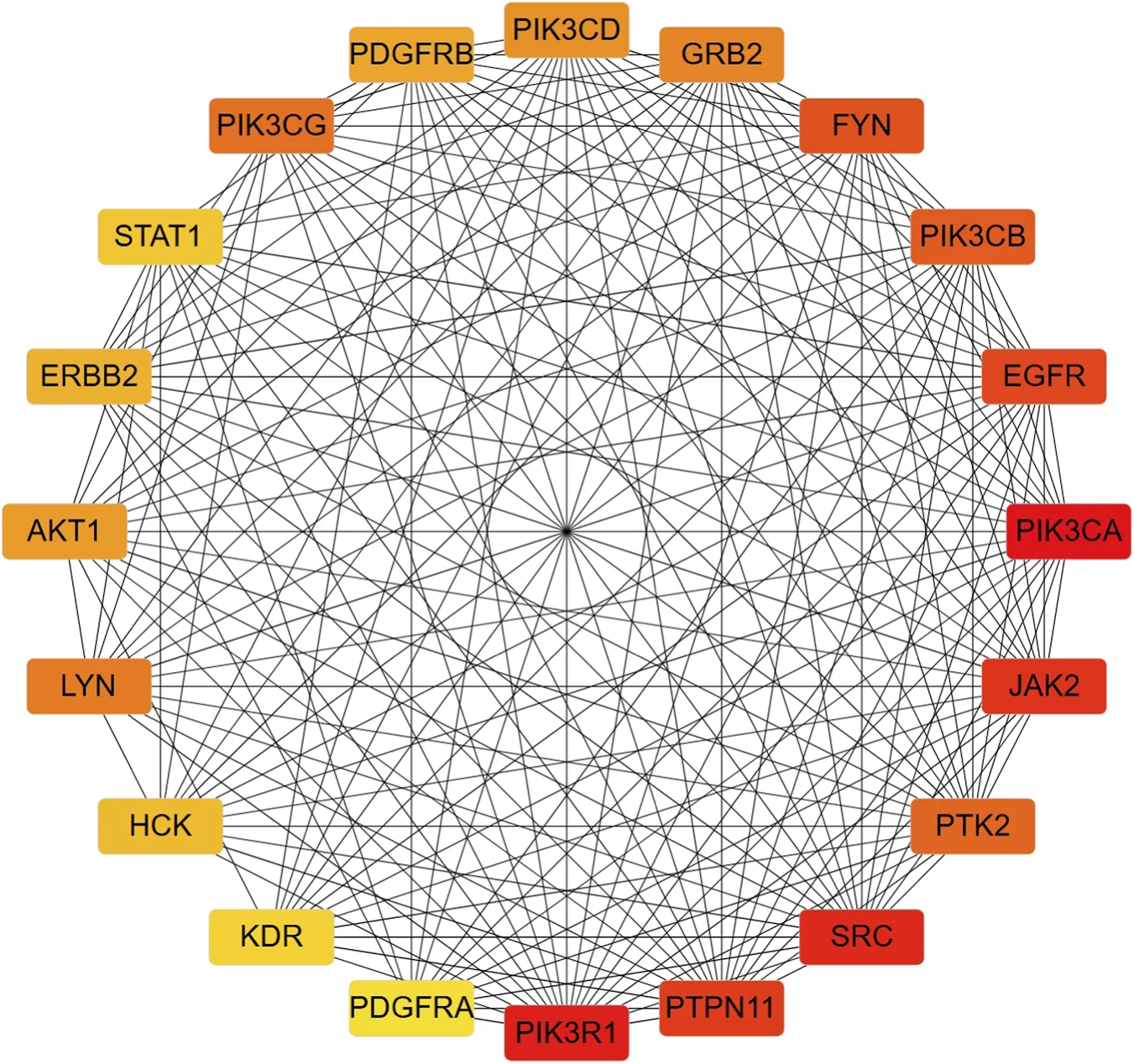
Identification of the top 20 core targets for HCQ-induced retinal toxicity. A sub-network of the top 20 hub proteins was extracted from the global PPI network using the Maximal Clique Centrality (MCC) algorithm in CytoHubba. Node colors ranging from yellow to dark red represent increasing MCC scores, with *PIK3CA*, *PIK3R1*, *SRC*, *JAK2*, and *PTPN11* identified as the most critical central nodes.

**TABLE 2:** Topological parameters of the top 20 hub targets.

Rank	Name	Score
1	PIK3CA	3703907836
2	PIK3R1	3703619096
3	SRC	3641397879
4	JAK2	3579471807
5	PTPN11	3540706392
6	EGFR	3520203217
7	FYN	3492734066
8	PIK3CB	3451717080
9	PTK2	3443781649
10	PIK3CG	3034931788
11	LYN	2276432362
12	GRB2	1910510300
13	PIK3CD	1788909360
14	AKT1	1314591204
15	PDGFRB	1261749822
16	ERBB2	1200026041
17	HCK	1091038368
18	STAT1	246283788
19	KDR	219952178
20	PDGFRA	170568756

### 3.3 Enrichment analyses for GO terms and KEGG pathways

To elucidate the systemic biological mechanisms underlying HCQ-induced retinal toxicity, we conducted Gene Ontology (GO) and Kyoto Encyclopedia of Genes and Genomes (KEGG) pathway enrichment analyses on the 480 intersecting core targets using the “clusterProfiler” package in software (v4.5.3) (R Core Team, 2023; Carlson, 2024).

Functional annotation revealed distinct clustering across the three GO categories (Figure 3A). Based on adjusted P-values, the top six significantly enriched biological processes (BP) were primarily associated with responses to external stimuli, including the response to lipopolysaccharide, response to molecules of bacterial origin, positive regulation of the MAPK cascade, cellular response to peptide hormone stimulus, response to xenobiotic stimuli, and response to peptide hormones. For cellular components (CC), the core targets were predominantly localized to secretory granule lumens, cytoplasmic vesicle lumens, vesicle lumens, membrane rafts, membrane microdomains, and the external side of the plasma membrane. Furthermore, the top six molecular functions (MF) were highly linked to kinase and receptor activities, specifically tyrosine kinase activity, protein tyrosine kinase activity, nuclear receptor activity, ligand-activated transcription factor activity, G protein-coupled peptide receptor activity, and peptide receptor activity.

**FIGURE 3:**
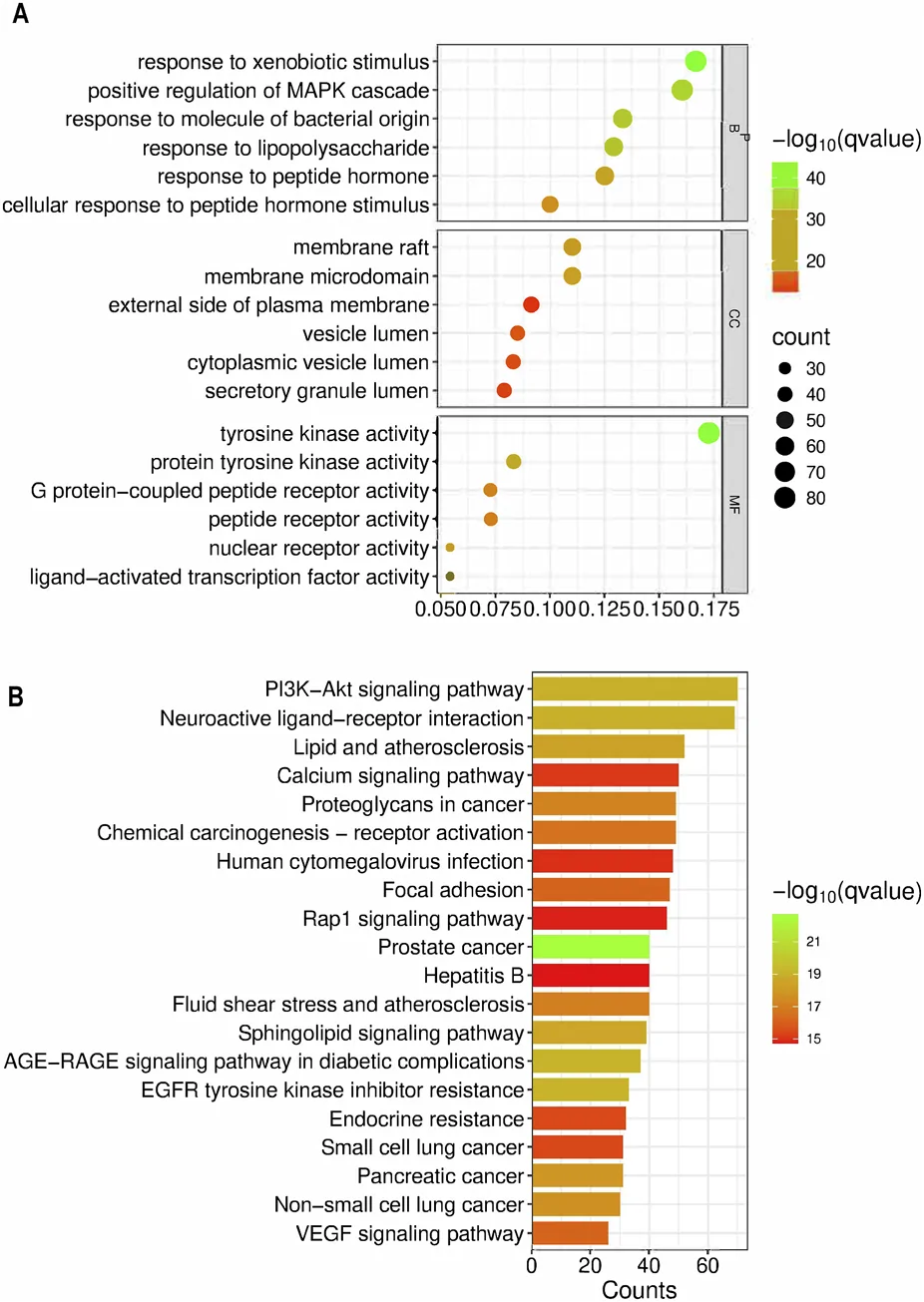
GO and KEGG pathway enrichment analyses of the core intersecting targets. **(A)** Bar chart illustrating the Gene Ontology (GO) functional enrichment analysis. The y-axis represents the significantly enriched GO terms, which are categorized into Biological Process (BP), Cellular Component (CC), and Molecular Function (MF). The x-axis indicates the gene ratio. The color gradient corresponds to the q-value. **(B)** Bubble chart depicting the top 20 enriched Kyoto Encyclopedia of Genes and Genomes (KEGG) pathways. The y-axis displays the pathway names, while the x-axis represents the gene ratio. The size of the bubbles reflects the number of enriched genes (Count), and the color gradient indicates the statistical significance (*q*-value). Notably, the PI3K-Akt signaling pathway emerged as the most significantly enriched pathway.

Similarly, KEGG pathway enrichment analysis identified 194 significantly enriched signaling cascades (adjusted *P* < 0.05). To better illustrate their enrichment significance and gene counts, the top 20 KEGG pathways were visualized in a bar chart (Figure 3B). These pathways predominantly included the PI3K-Akt signaling pathway, neuroactive ligand-receptor interactions, lipid and atherosclerosis, calcium signaling pathways, proteoglycans in cancer, chemical carcinogenesis-receptor activation, and human cytomegalovirus infection. Most notably, the PI3K-Akt signaling pathway emerged as the most robustly enriched pathway with the highest gene count, suggesting that the perturbation of this crucial survival and metabolic axis is the primary driving mechanism of HCQ-induced retinal toxicity.

### 3.4 Molecular docking suggests potential interactions between HCQ and candidate hub proteins

Molecular docking was performed to evaluate the potential interactions between HCQ and the five candidate hub proteins, including PIK3CA (PDB ID: 8EXL), PIK3R1 (PDB ID: 7RNS), SRC (PDB ID: 2BDJ), JAK2 (PDB ID: 7RN6), and PTPN11 (PDB ID: 3B7O). The selected structures represent experimentally determined human protein structures corresponding to the respective targets.

AutoDock 4.2.6 analysis yielded predicted docking energies ranging from −2.80 to −6.14 kcal/mol. Among the five proteins, HCQ showed the lowest docking energy with PIK3R1 (−6.14 kcal/mol), followed by PIK3CA (−5.09 kcal/mol), SRC (−4.59 kcal/mol), JAK2 (−2.89 kcal/mol), and PTPN11 (−2.80 kcal/mol). These results indicate that HCQ could adopt predicted docking conformations within the selected binding regions of the candidate proteins, although the docking scores varied among targets.

For each protein, the pose with the lowest AutoDock-estimated docking energy within the selected binding region was used for structural analysis. Three-dimensional protein–HCQ complexes were visualized using PyMOL 3.0.3, and the corresponding protein–ligand interactions, including hydrogen bonds and surrounding interacting residues, were further examined using Schrödinger Maestro 2021 (Figure 4). The AutoDock-derived energies were interpreted as computational docking scores rather than experimentally determined binding affinities.

**FIGURE 4:**
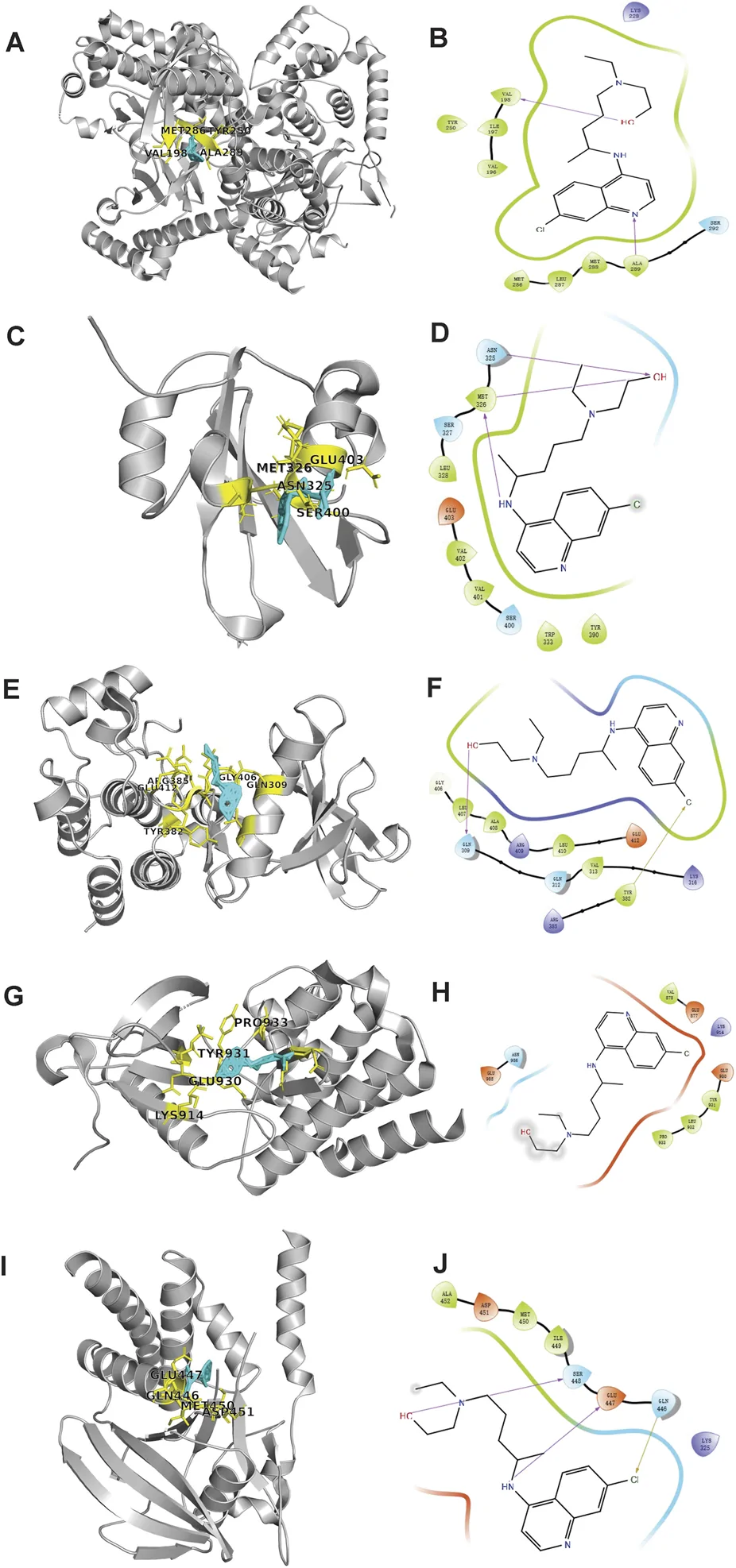
Predicted binding modes of HCQ with the five candidate hub proteins. Three-dimensional docking poses and corresponding twodimensional interaction diagrams are shown for HCQ with PIK3CA (PDB ID: 8EXL; **(A,B)**; predicted binding energy: −5.09 kcal/mol), PIK3R1 (PDB ID: 7RNS; **(C,D)**; −6.14 kcal/mol), SRC (PDB ID: 2BDJ; **(E,F)**; −4.59 kcal/mol), JAK2 (PDB ID: 7RN6; **(G,H)**; −2.89 kcal/mol), and PTPN11 (PDB ID: 3B7O; **(I,J)**; −2.80 kcal/mol). Panels **(A,C,E,G,I)** show the predicted three-dimensional binding conformations of HCQ within the selected protein binding regions, with surrounding amino acid residues indicated. Panels **(B,D,F,H,J)** show the corresponding two-dimensional interaction maps. The reported energies are AutoDock-derived docking estimates.

### 3.5 HCQ induces concentration-dependent cytotoxicity in ARPE-19 cells

To evaluate the cytotoxic effects of HCQ in retinal pigment epithelial cells, ARPE-19 cells were exposed to increasing concentrations of HCQ for 24 h. CCK-8 assays showed a progressive reduction in cell viability with increasing HCQ concentrations (Figure 5A). Nonlinear regression using a log (inhibitor) versus normalized response model with a variable slope yielded a 24-h IC_50_ of 59.51 μM (95% CI: 56.08–63.06 μM; R^2^ = 0.9686), with a fitted Hill slope of −1.404 (Figure 5B). The analysis was based on three independent biological experiments, with five technical replicate wells per concentration in each experiment. Based on the concentration–response relationship and the estimated IC_50_, 15, 30, and 60 μM HCQ were selected as representative low, intermediate, and high exposure concentrations, respectively, for subsequent mechanistic experiments.

**FIGURE 5:**
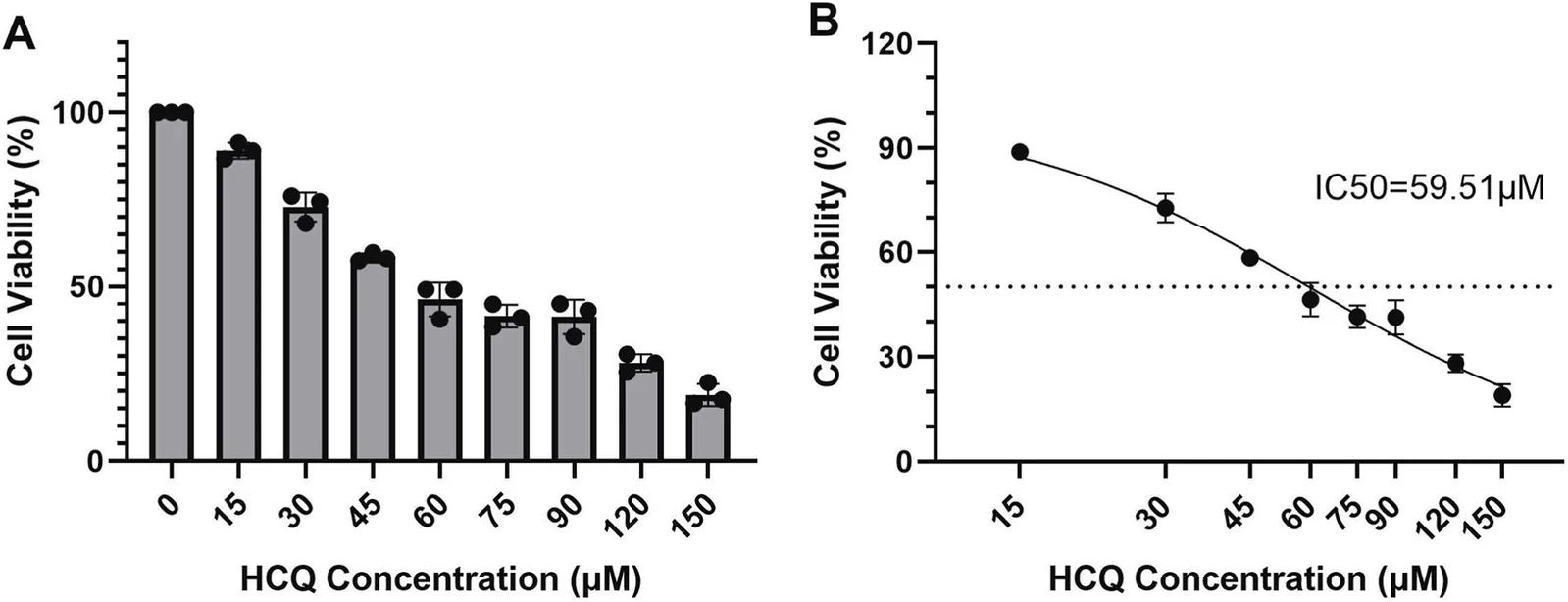
HCQ induces concentration-dependent cytotoxicity in ARPE-19 cells. **(A)** Cell viability of ARPE-19 cells following 24-h exposure to increasing concentrations of HCQ (0–150 μM), as determined by the CCK-8 assay. Cell viability progressively decreased with increasing HCQ concentrations. **(B)** Concentration–response curve for HCQ-induced cytotoxicity. A four-parameter logistic nonlinear regression model yielded an estimated 24-h IC_50_ value of 59.51 μM. Data were obtained from at least three independent biological experiments (n ≥ 3). Multiple technical replicates within each independent experiment were averaged to generate a single value for each concentration. Data are presented as mean ± SD.

### 3.6 HCQ increases apoptosis in ARPE-19 cells in a concentration-associated manner

To further characterize HCQ-induced cytotoxicity, apoptosis was assessed by Annexin V-FITC/PI flow cytometry in ARPE-19 cells exposed to 0, 15, 30, or 60 μM HCQ for 24 h. Representative flow cytometric plots showed a progressive shift from the Annexin V^−^/PI^−^ viable population toward Annexin V-positive populations with increasing HCQ concentrations (Figures 6A–D). Quantitative analysis of three independent biological experiments showed an increase in total apoptotic cells, defined as the sum of early apoptotic (Annexin V^+^/PI^−^) and late apoptotic (Annexin V^+^/PI^+^) populations, following HCQ treatment compared with the vehicle control (Figure 6E). The apoptotic fraction increased with increasing HCQ concentrations and was highest at 60 μM. These findings demonstrate that the HCQ-induced reduction in ARPE-19 cell viability was accompanied by a concentration-associated increase in apoptosis.

**FIGURE 6:**
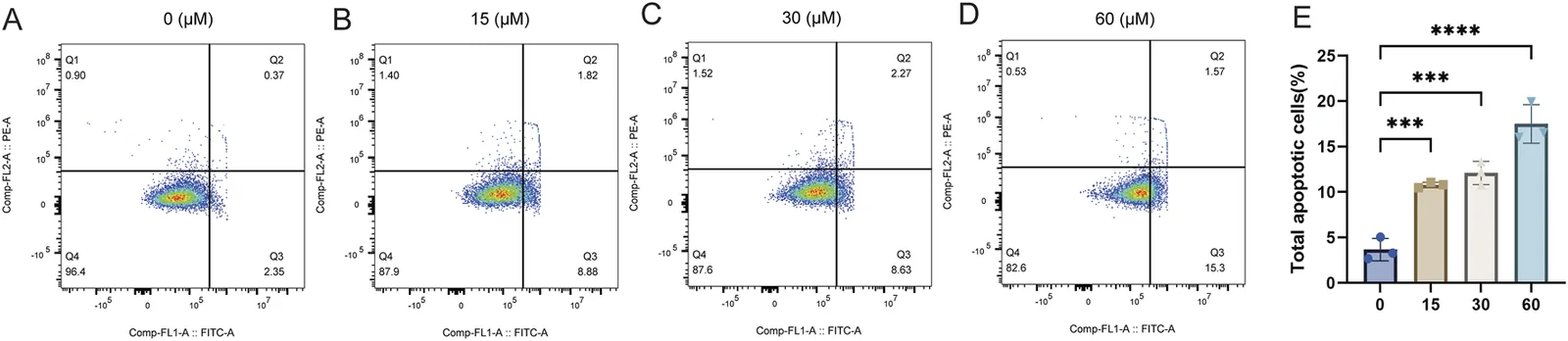
HCQ increases apoptosis in ARPE-19 cells in a concentration-dependent manner. **(A–D)** Representative Annexin V–FITC/PI flow cytometry plots of ARPE-19 cells following 24-h treatment with 0, 15, 30, or 60 μM HCQ, respectively. Q4 represents viable cells (Annexin V^−^/PI^−^), Q3 early apoptotic cells (Annexin V^+^/PI^−^), Q2 late apoptotic cells (Annexin V^+^/PI^+^), and Q1 PI-positive/Annexin V-negative cells. **(E)** Quantification of total apoptotic cells, calculated as the sum of early and late apoptotic populations (Q3 + Q2). HCQ treatment increased the proportion of apoptotic cells with increasing concentrations. Data are presented as mean ± SD from three independent biological experiments (n = 3). ns, not significant; *P < 0.05, **P < 0.01, ***P < 0.001, and ****P < 0.0001 versus the control group.

### 3.7 HCQ induces gene-specific transcriptional alterations in candidate hub genes

To determine whether the candidate hub genes identified through network toxicology were transcriptionally altered following HCQ exposure, the mRNA expression levels of *PIK3CA*, *PIK3R1*, *SRC*, *JAK2*, and *PTPN11* were examined in ARPE-19 cells after 24 h treatment with 0, 15, 30, or 60 μM HCQ. Gene expression was normalized using *GAPDH* and *RPLP0* as reference genes. The five genes exhibited distinct concentration-related transcriptional responses to HCQ exposure (Figures 7A–E).

**FIGURE 7:**
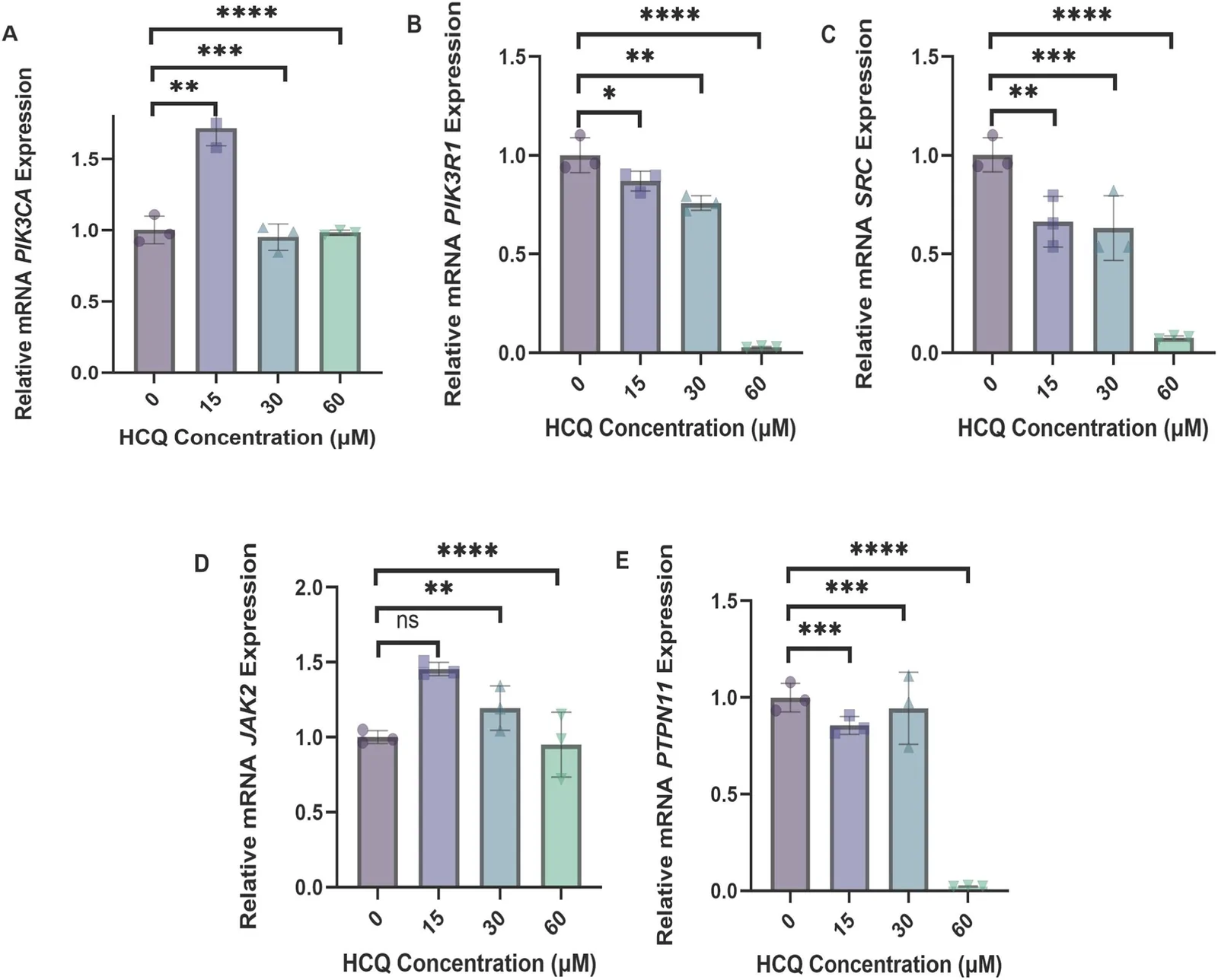
HCQ induces concentration-dependent and gene-specific transcriptional changes in predicted hub genes in ARPE-19 cells. ARPE-19 cells were treated with HCQ at 0, 15, 30, or 60 μM for 24 h. Relative mRNA expression levels of the five predicted hub genes, **(A)** *PIK3CA*, **(B)** *PIK3R1*, **(C)** *SRC*, **(D)** *JAK2*, and **(E)** *PTPN11*, were determined by qRT-PCR using *GAPDH* and *RPLP0* as reference genes for normalization. Data are presented as mean ± SD from three independent biological experiments (n = 3). Statistical significance was assessed using one-way ANOVA followed by Dunnett’s multiple-comparisons test, with each HCQ-treated group compared with the control group (0 μM). ns, not significant; *P < 0.05, **P < 0.01, ***P < 0.001, and ****P < 0.0001 versus the control group.

*PIK3CA* expression was increased at 15 μM HCQ and subsequently returned toward the control level at 30 and 60 μM (Figure 7A). In contrast, *PIK3R1* expression showed an overall decrease with increasing HCQ concentrations, with the greatest reduction observed at 60 μM (Figure 7B). SRC exhibited a similar overall pattern, with reduced expression at 15 and 30 μM and a more pronounced decrease at 60 μM (Figure 7C).

*JAK2* exhibited a non-monotonic transcriptional response, with no significant change at 15 μM, increased expression at 30 μM, and a decline at 60 μM (Figure 7D). *PTPN11* expression was reduced at 15 μM, showed a partial recovery at 30 μM, and was markedly suppressed at 60 μM HCQ (Figure 7E).

Collectively, these findings demonstrate that HCQ induces gene-specific and concentration-dependent transcriptional alterations in the predicted hub genes rather than uniform suppression of all five targets. The distinct response patterns among individual genes indicate differential transcriptional regulation of PI3K/AKT-associated signaling components during HCQ exposure.

### 3.8 HCQ reduces the abundance of predicted hub proteins in ARPE-19 cells

To determine whether HCQ exposure was associated with changes in the protein abundance of the predicted hub targets, PIK3CA, PIK3R1, SRC, JAK2, and PTPN11 were examined in ARPE-19 cells after 24 h treatment with 0, 15, 30, or 60 μM HCQ. Western blot analysis showed that the abundance of all five PI3K/AKT-associated hub proteins generally decreased with increasing HCQ concentrations, although the magnitude of the reduction varied among individual proteins (Figures 8A–E). The reductions were generally more apparent at 30 and 60 μM HCQ.

**FIGURE 8:**
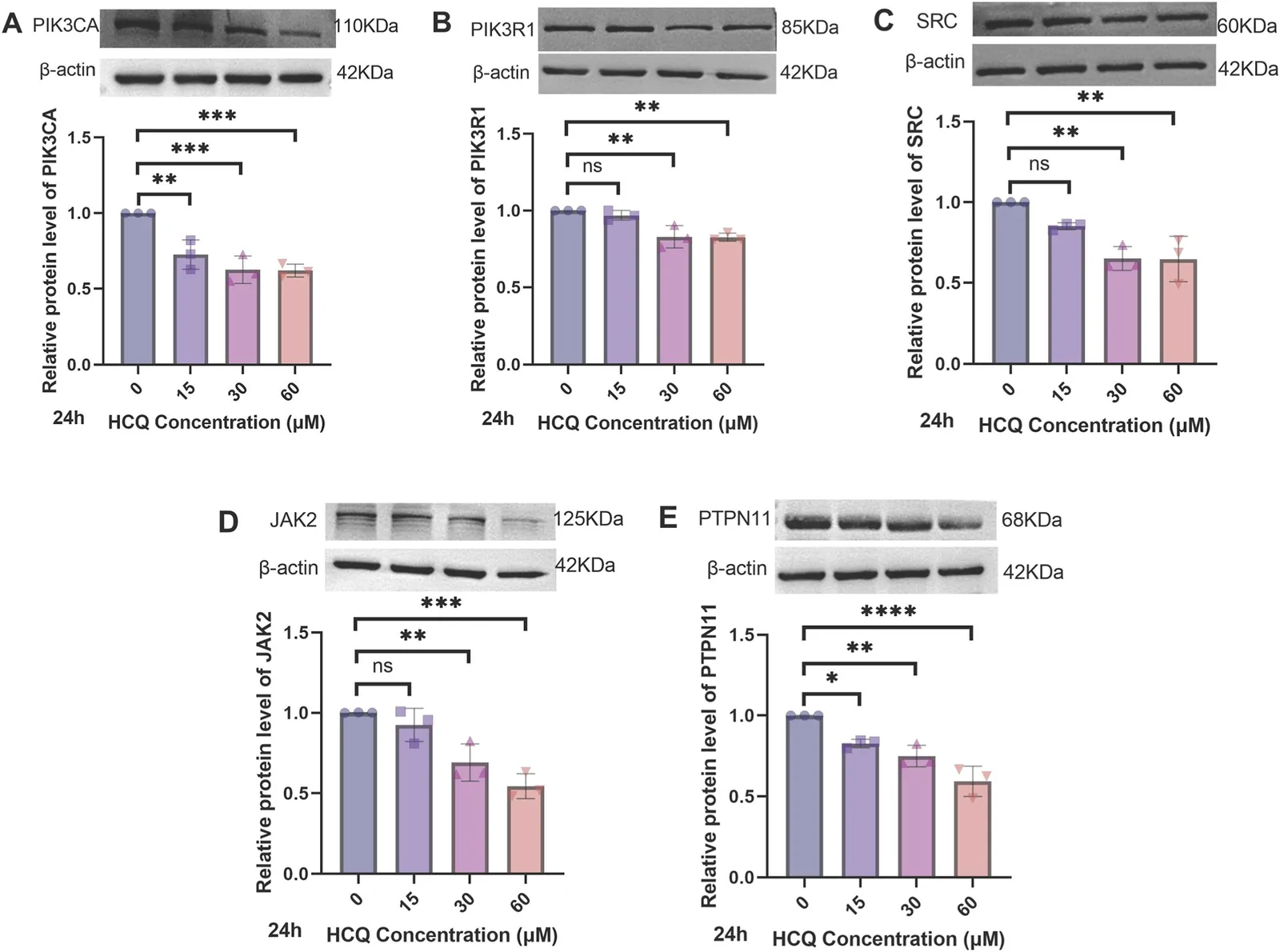
HCQ reduces the abundance of network toxicology-predicted hub proteins in ARPE-19 cells after 24 h exposure. ARPE-19 cells were treated with HCQ at 0, 15, 30, or 60 μM for 24 h. Representative Western blot images and quantitative analyses are shown for **(A)** PIK3CA, **(B)** PIK3R1, **(C)** SRC, **(D)** JAK2, and **(E)** PTPN11. Protein levels were normalized to β-actin. HCQ treatment generally reduced the abundance of the five candidate hub proteins with increasing concentrations. Data are presented as mean ± SD from three independent biological experiments (n = 3). Statistical significance was assessed using one-way ANOVA followed by Dunnett’s multiple-comparisons test, with each HCQ-treated group compared with the control group (0 μM). ns, not significant; *P < 0.05, **P < 0.01, ***P < 0.001, and ****P < 0.0001 versus the control group.

Notably, the protein-level changes did not fully parallel the corresponding transcriptional responses. Nevertheless, the overall reduction in the abundance of multiple candidate hub proteins following HCQ exposure indicates multilevel dysregulation of PI3K/AKT-associated signaling components in ARPE-19 cells.

### 3.9 HCQ induces concentration- and time-dependent alterations in AKT phosphorylation

Because AKT phosphorylation represents a dynamic readout of PI3K/AKT-associated signaling, changes in the p-AKT/AKT ratio were examined under different HCQ concentrations and exposure durations. Following acute 1-h exposure, 15 μM HCQ did not significantly alter the p-AKT/AKT ratio, whereas 30 and 60 μM HCQ significantly increased AKT phosphorylation compared with the vehicle control (Figure 9A).

**FIGURE 9:**
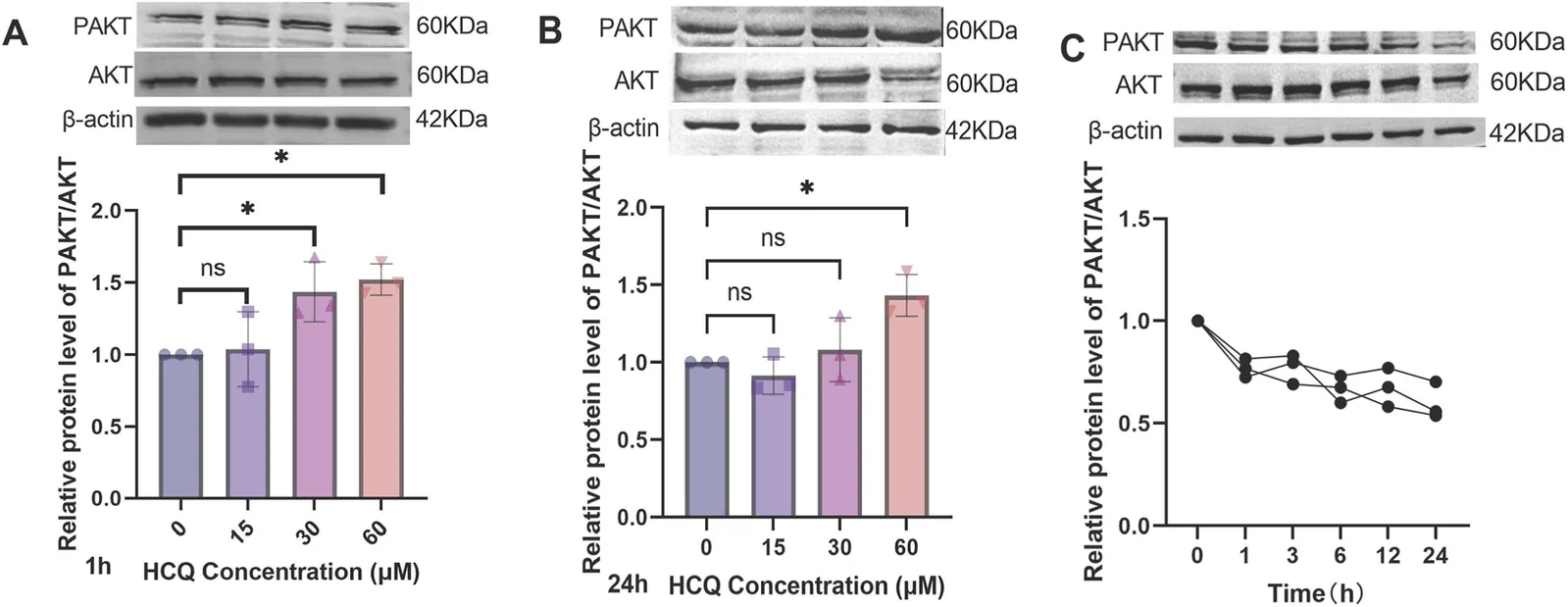
HCQ induces concentration- and time-dependent alterations in AKT phosphorylation in ARPE-19 cells. ARPE-19 cells were treated with HCQ, and AKT phosphorylation was evaluated by Western blotting. **(A)** Representative immunoblots and quantitative analysis of the p-AKT/AKT ratio after 1 h exposure to HCQ at 0, 15, 30, or 60 μM. **(B)** Representative immunoblots and quantitative analysis of the p-AKT/AKT ratio after 24 h exposure to the same HCQ concentrations. **(C)** Time-course analysis of the p-AKT/AKT ratio in ARPE-19 cells treated with 30 μM HCQ for 0, 1, 3, 6, 12, and 24 h p-AKT levels were normalized to total AKT, and β-actin was used as the loading control. Data in panels A and B are presented as mean ± SD from three independent biological experiments (n = 3). Individual biological replicates are shown in panel **(C)** ns, not significant; *P < 0.05, **P < 0.01, ***P < 0.001, and ****P < 0.0001 versus the control group.

After 24 h of exposure, the phosphorylation response differed from that observed at 1 h. The p-AKT/AKT ratio at 15 and 30 μM did not differ significantly from that of the vehicle control, indicating that the increase observed at 30 μM after acute exposure was not maintained at the later time point. In contrast, p-AKT/AKT remained significantly elevated following treatment with 60 μM HCQ (Figure 9B).

To further characterize the temporal response, an independent time-course experiment was performed using 30 μM HCQ. Across the 0–24 h time course, the p-AKT/AKT ratio showed an overall downward trajectory during prolonged HCQ exposure, with the lowest level observed at 24 h (Figure 9C). Collectively, these results indicate that HCQ-associated changes in AKT phosphorylation depend on both concentration and exposure duration, demonstrating dynamic regulation rather than sustained unidirectional activation or inhibition of AKT signaling.

### 3.10 SC79 enhances AKT phosphorylation and partially attenuates HCQ-induced apoptosis in ARPE-19 cells

To determine an appropriate working concentration of SC79 for the rescue experiments, ARPE-19 cells were treated with 0, 2.5, 5, 7.5, or 10 μg/mL SC79. No significant reduction in cell viability was observed across the tested concentration range (Figure 10A). Analysis of AKT phosphorylation showed a variable response across SC79 concentrations, with the highest mean p-AKT/AKT ratio observed at 5 μg/mL, although inter-experimental variability was evident (Figure 10B). Based on the absence of a significant reduction in cell viability and the observed AKT phosphorylation response, 5 μg/mL SC79 was selected for subsequent rescue experiments.

**FIGURE 10:**
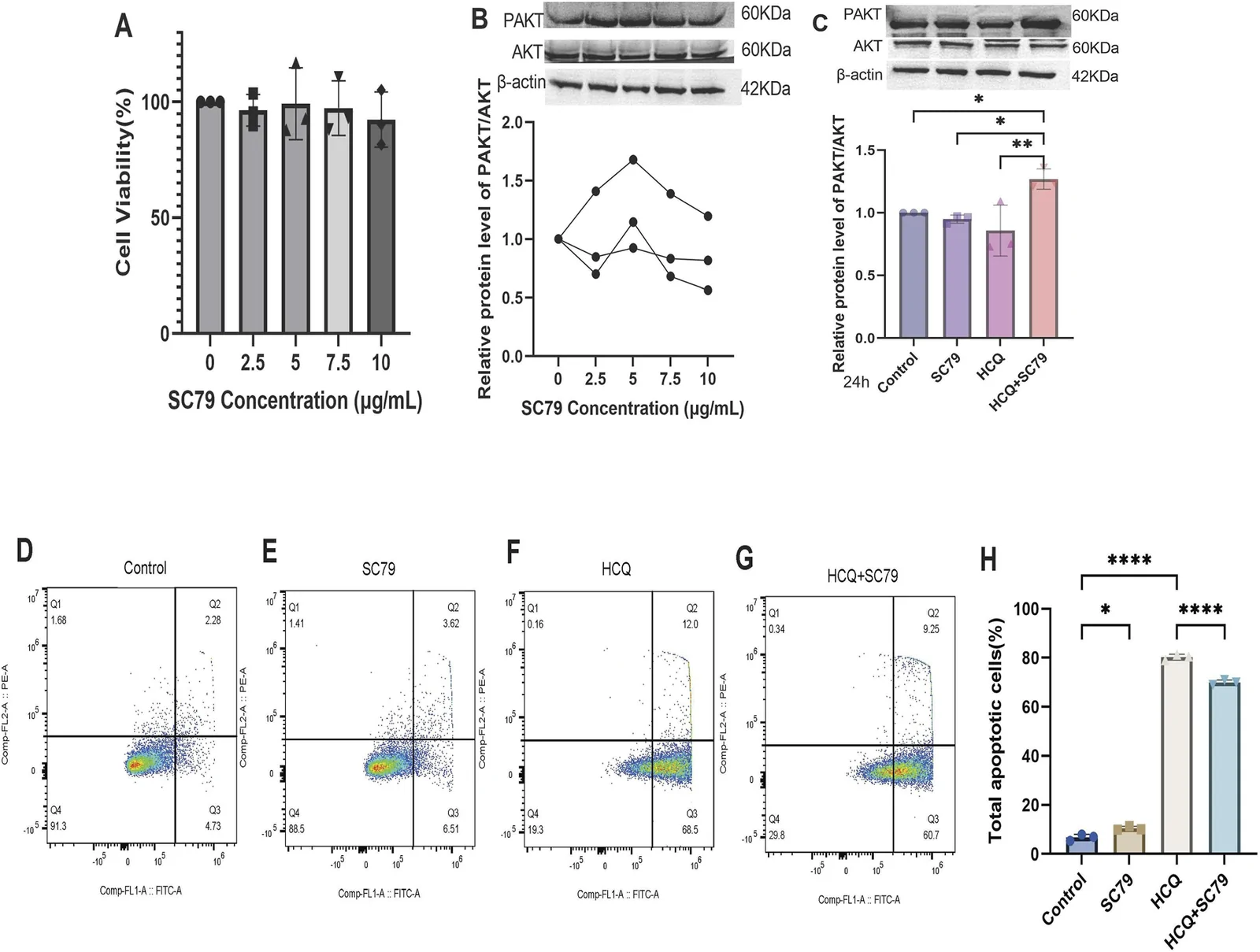
SC79 activates AKT signaling and partially attenuates HCQ-induced apoptosis in ARPE-19 cells. **(A)** Cell viability of ARPE-19 cells following treatment with increasing concentrations of SC79 (0, 2.5, 5, 7.5, and 10 μg/mL), as assessed by the CCK-8 assay. SC79 did not significantly reduce cell viability within the tested concentration range. **(B)** Representative Western blots and analysis of the p-AKT/AKT ratio following treatment with the indicated concentrations of SC79. A concentration of 5 μg/mL showed the highest mean p-AKT/AKT ratio among the tested concentrations and was therefore selected for subsequent rescue experiments. For the rescue experiments, ARPE-19 cells were treated with HCQ (60 μM), SC79 (5 μg/mL), or their combination for 24 h. **(C)** Representative Western blots and quantitative analysis of the p-AKT/AKT ratio in the Control, SC79, HCQ, and HCQ + SC79 groups. Co-treatment with SC79 significantly increased the p-AKT/AKT ratio compared with HCQ treatment alone. p-AKT levels were normalized to total AKT, and β-actin served as the loading control. **(D–G)** Representative Annexin V–FITC/PI flow cytometry plots for the Control, SC79, HCQ, and HCQ + SC79 groups, respectively. Q4 represents viable cells (Annexin V^−^/PI^−^), Q3 early apoptotic cells (Annexin V^+^/PI^−^), Q2 late apoptotic cells (Annexin V^+^/PI^+^), and Q1 PI-positive/Annexin V-negative cells. **(H)** Quantification of total apoptotic cells, calculated as the sum of early and late apoptotic populations (Q3 + Q2). SC79 co-treatment partially reduced HCQ-induced apoptosis. Data are presented as mean ± SD from three independent biological experiments (n = 3), except that individual biological replicates are shown in panel **(B)**. Statistical significance is indicated by the brackets shown in the figure. ns, not significant; *P < 0.05, **P < 0.01, ***P < 0.001, and ****P < 0.0001 versus the control group.

For the rescue experiments, ARPE-19 cells were pretreated with SC79 (5 μg/mL) for 1 h before exposure to HCQ (60 μM) for 24 h. The p-AKT/AKT ratio was significantly higher in the HCQ + SC79 group than in the HCQ-alone group (Figure 10C), indicating that SC79 increased AKT phosphorylation under HCQ exposure.

The functional effect of SC79 was further assessed by Annexin V-FITC/PI flow cytometry (Figures 10D–G). HCQ significantly increased the proportion of total apoptotic cells compared with the vehicle control (Figure 10H). SC79 pretreatment significantly reduced the total apoptotic fraction compared with HCQ treatment alone, although apoptosis remained significantly higher than that in the vehicle control, indicating partial attenuation of HCQ-induced apoptosis. SC79 alone produced a modest but statistically significant increase in apoptosis compared with the vehicle control. Collectively, these findings show that pharmacological activation of AKT with SC79 enhances AKT phosphorylation under HCQ exposure and partially attenuates HCQ-induced apoptosis, supporting a functional involvement of AKT signaling in the cellular response to HCQ.

## 4 Discussion

### 4.1 HCQ-associated dysregulation of an upstream PI3K/AKT-related signaling network

Hydroxychloroquine (HCQ) is widely used for the long-term treatment of autoimmune and rheumatologic disorders; however, retinal toxicity remains an important adverse effect that may cause irreversible visual impairment. The risk of HCQ retinopathy is influenced by treatment duration, cumulative exposure, daily dose, and individual susceptibility, including renal dysfunction and other clinical risk factors (Jorge et al., 2024; Marmor et al., 2026). Recent clinical imaging studies have also identified alterations involving the retinal pigment epithelium (RPE), ellipsoid zone (EZ), and external limiting membrane (ELM) in HCQ-treated patients, supporting the involvement of both RPE and outer-retinal structures in the toxic process (Yucel Gencoglu et al., 2024).

In the present study, we integrated network toxicology, molecular docking, and experimental validation in ARPE-19 cells to investigate signaling mechanisms associated with HCQ-induced cytotoxicity. Network analysis identified PIK3CA, PIK3R1, SRC, JAK2, and PTPN11 as candidate hub proteins linked to PI3K/AKT-associated signaling. Subsequent cellular experiments demonstrated concentration-dependent reductions in cell viability, increased apoptosis detected by Annexin V/PI staining, altered transcription of the predicted hub genes, reduced abundance of multiple corresponding proteins after 24 h HCQ exposure, and time-dependent alterations in AKT phosphorylation. PI3K/AKT signaling is a central regulator of cellular survival, growth, metabolism, and stress responses (Manning and Cantley, 2007; Glaviano et al., 2023), and previous studies in RPE cells have also linked PI3K/AKT-related signaling to oxidative and metabolic stress responses (Chen et al., 2019).

Previous studies indicate that HCQ-associated retinal injury involves multiple cellular processes. HCQ can interfere with lysosomal activity and autophagic processing (Schrezenmeier and Dörner, 2020), while Mondal et al. demonstrated early retinal abnormalities together with alterations in the autophagosome–lysosomal pathway and sphingolipid metabolism (Mondal et al., 2022). In ARPE-19 cells, Ertuğrul et al. further showed that HCQ exposure was associated with increased oxidative stress, mitochondrial dysfunction, apoptosis, and cell death (Ertuğrul et al., 2023). These observations suggest that lysosomal dysfunction, autophagic disturbance, oxidative stress, and survival signaling may interact during HCQ-induced cellular injury. Our findings extend these established mechanisms by identifying PI3K/AKT-associated signaling as an additional functionally relevant component of the HCQ response.

Molecular docking further suggested that HCQ could adopt energetically favorable conformations within selected binding regions of the five candidate proteins, although the predicted docking energies varied among targets. These computational results provide structural hypotheses for potential HCQ–protein interactions. Accordingly, PIK3CA, PIK3R1, SRC, JAK2, and PTPN11 should be regarded as candidate HCQ-associated regulators requiring further target-engagement validation.

### 4.2 Concentration-dependent and gene-specific transcriptional dysregulation

The transcriptional responses to HCQ were concentration dependent but differed among the five candidate hub genes. *PIK3CA* showed increased expression at 15 μM, whereas *PIK3R1* and *SRC* exhibited an overall reduction with increasing HCQ concentrations. *JAK2* displayed a non-monotonic response, with no significant change at 15 μM, increased expression at 30 μM, and a subsequent decline at 60 μM. *PTPN11* was reduced at 15 μM, showed partial recovery at 30 μM, and was markedly suppressed at 60 μM. These heterogeneous responses indicate that HCQ does not uniformly suppress the predicted signaling network but instead induces gene-specific transcriptional regulation.

Such nonuniform responses may reflect differences in the sensitivity and regulatory roles of individual signaling components during cellular stress. The increase in selected transcripts at lower or intermediate HCQ concentrations may represent a compensatory component of the cellular stress response, whereas the marked suppression of several hub genes at higher concentrations suggests that this regulatory capacity becomes altered as cellular stress increases. Stress-adaptive mechanisms have also been described in retinal disease biology, providing a broader context for transient compensatory responses under cellular stress (Lin et al., 2025).

Overall, these findings are more consistent with a complex concentration-dependent transcriptional response than with uniform pathway inhibition. Given that HCQ also affects lysosomal function, autophagic processing, and lipid metabolism (Mondal et al., 2022), the transcriptional changes observed here likely represent one component of a broader cellular stress response. Notably, these transcriptional changes were not fully paralleled by the corresponding protein-level responses, suggesting that HCQ-associated regulation of this signaling network may occur at multiple molecular levels.

### 4.3 Time-dependent AKT signaling suggests dynamic adaptive regulation during HCQ exposure

A particularly important finding of the present study was the dynamic regulation of AKT phosphorylation during HCQ exposure. Short-term treatment produced an increase in p-AKT/AKT at higher HCQ concentrations, whereas the 30 μM time-course experiment showed an overall downward trajectory in AKT phosphorylation during prolonged exposure. These findings indicate that AKT signaling is regulated in both a time- and concentration-dependent manner rather than undergoing uniform inhibition.

The early increase in p-AKT may represent a rapid adaptive survival response to HCQ-induced stress. AKT is a highly dynamic signaling node involved in cellular survival, metabolism, and resistance to apoptotic stimuli (Manning and Toker, 2017). Transient activation of AKT has also been observed under acute cellular stress. For example, Ahmad et al. reported early AKT activation following hyperoxic exposure and demonstrated a protective role of AKT signaling against cellular injury (Ahmad et al., 2006). Although this model differs from HCQ-treated RPE cells, it supports the broader concept that acute stress can transiently activate AKT-dependent survival signaling.

At 24 h, the abundance of PIK3CA, PIK3R1, SRC, JAK2, and PTPN11 generally decreased with increasing HCQ concentrations. These protein-level changes did not fully parallel the corresponding transcriptional responses, suggesting that regulation may occur at multiple molecular levels. In parallel, the 30 μM time-course experiment indicated that AKT phosphorylation was not maintained during prolonged exposure. Together, these findings suggest that HCQ induces dynamic and multilevel dysregulation of PI3K/AKT-associated signaling rather than uniform suppression of the pathway.

### 4.4 Pharmacological activation of AKT partially attenuates HCQ-induced apoptosis

The addition of the AKT activator SC79 provided functional evidence supporting the involvement of AKT signaling in the cellular response to HCQ. SC79 is a small-molecule AKT activator that promotes cytosolic AKT phosphorylation and has been shown to enhance cell survival under stress conditions (Jo et al., 2012). During preliminary concentration screening, SC79 did not significantly reduce ARPE-19 cell viability within the tested concentration range, while 5 μg/mL showed the highest mean p-AKT/AKT ratio among the tested concentrations and was therefore selected for subsequent rescue experiments.

To evaluate whether pharmacological enhancement of AKT signaling could modify the cellular response to HCQ, ARPE-19 cells were pretreated with SC79 before HCQ exposure. SC79 pretreatment significantly increased the p-AKT/AKT ratio compared with HCQ treatment alone. Consistent with this enhancement of AKT phosphorylation, Annexin V/PI flow cytometry showed that SC79 pretreatment partially reduced HCQ-induced apoptosis. These findings provide functional evidence that modulation of AKT signaling influences the apoptotic response to HCQ.

HCQ-associated retinal toxicity has also been linked to lysosomal dysfunction, impaired autophagic processing, and other cellular disturbances (Paniri et al., 2020; Mondal et al., 2022), suggesting that PI3K/AKT signaling may interact with additional stress-response pathways. The SC79 rescue findings complement the concentration- and time-dependent AKT phosphorylation data and further support a functional role for AKT signaling in the cellular response to HCQ. Nevertheless, potential off-target effects of SC79 cannot be excluded, and genetic approaches will be required to establish pathway-specific causality more definitively.

### 4.5 Clinical implications, limitations, and future perspectives

The present findings support an important role for PI3K/AKT-associated signaling in HCQ-induced cellular stress and apoptosis. Clinically, HCQ-associated ocular toxicity is predominantly related to long-term exposure, with cumulative dose and treatment duration contributing importantly to its development (Liu et al., 2024). Clinical OCT studies have also demonstrated structural alterations involving the RPE and ellipsoid zone in patients with HCQ retinopathy (He et al., 2024), providing clinical context for the RPE-related cellular responses observed in the present study. The ARPE-19 model provides a controlled experimental system for investigating HCQ-induced RPE signaling responses, while the chronic and multicellular nature of clinical retinopathy should be considered when extending these mechanistic observations to patients.

Several limitations should be acknowledged. First, ARPE-19 cells provide a widely used and experimentally tractable human RPE-derived model for investigating cellular responses and molecular mechanisms. Under appropriate culture conditions, these cells can reproduce several characteristic features of RPE cells (Bharti et al., 2022). However, their phenotype is influenced by culture conditions and does not fully reproduce the differentiated state and tissue environment of native human RPE. Second, although Annexin V/PI analysis confirmed increased apoptosis and SC79 partially attenuated this phenotype, additional downstream apoptotic and stress-related mechanisms remain to be characterized. Future studies using genetic manipulation, primary or differentiated RPE models, retinal organoids, and chronic *in vivo* models will be valuable for further validating and extending the present findings.

Overall, the present study supports an important role for PI3K/AKT-associated signaling in HCQ-induced cytotoxicity in ARPE-19 cells. The findings indicate that HCQ perturbs this signaling network in a concentration- and time-dependent manner and that pharmacological modulation of AKT signaling influences the apoptotic response to HCQ. Together, these observations provide a mechanistic framework for understanding HCQ-induced RPE cellular injury and support further investigation of PI3K/AKT-associated signaling in HCQ-associated retinal toxicity.

## 5 Conclusion

In conclusion, this study integrates network toxicology, molecular docking, and experimental validation to identify a PI3K/AKT-associated signaling network involved in HCQ-induced cytotoxicity in ARPE-19 cells. HCQ induced concentration- and time-dependent alterations in upstream signaling components and AKT phosphorylation, accompanied by reduced cell viability and increased apoptosis. The dynamic changes in AKT phosphorylation, including early activation followed by impaired maintenance during sustained exposure, suggest a transition from adaptive signaling to compromised survival signaling. Importantly, pharmacological reactivation of AKT with SC79 partially attenuated HCQ-induced apoptosis, providing functional support for the involvement of AKT signaling in this process. Collectively, these findings provide a mechanistic framework for understanding HCQ-induced RPE cellular injury and support further investigation of PI3K/AKT-associated signaling as a functionally relevant pathway in HCQ cytotoxicity.

## Data Availability

The dataset containing the 480 intersecting targets identified between HCQ-associated targets and retinal toxicity-associated targets is publicly available in Mendeley Data at doi: 10.17632/4k9p437cw8.1. Additional data supporting the findings of this study are available from the corresponding author upon reasonable request.
